# Submandibular gland cystadenocarcinoma with mucinous adenocarcinoma-like areas: a case report

**DOI:** 10.1186/1746-1596-9-87

**Published:** 2014-04-28

**Authors:** Chun Zhang, Chunyan Chen, Jianrong Wang

**Affiliations:** 1 Department Pathology, Armed Police Corps Hospital of Chongqing, Chongqing 400061, China

**Keywords:** Cystadenocarcinoma, Submandibular gland, Cysts, Papillary, Mucus-like materials

## Abstract

**Abstract:**

Cystadenocarcinoma is primarily characterized by cystic structures of varying sizes, that are lined by epithelial cells. As a rare neoplasm, only four cases of cystadenocarcinoma of submandibular glands have been previously reported. Herein, we reported a unique case of submandibular gland cystadenocarcinoma in a 44-year-old man. By in large, this case had typical morphologic cystadenocarcinoma features. However, mucinous adenocarcinoma-like areas were additionally observed in the tumor tissues. This case was initially misdiagnosed as mucinous adenocarcinoma due to the low incidence of submandibular gland cystadenocarcinoma and the presentation of mucinous adenocarcinoma-like areas in the tumor tissues. Histopathology of additional tumor tissues revealed that mucinous adenocarcinoma-like areas accounted for only a small percentage of the tumor tissues, confirming the submandibular gland cystadenocarcinoma diagnosis.

**Virtual slides:**

The virtual slide(s) for this article can be found here: http://www.diagnosticpathology.diagnomx.eu/vs/1387916949121142

## Letter to the editor

**Figure 1:**
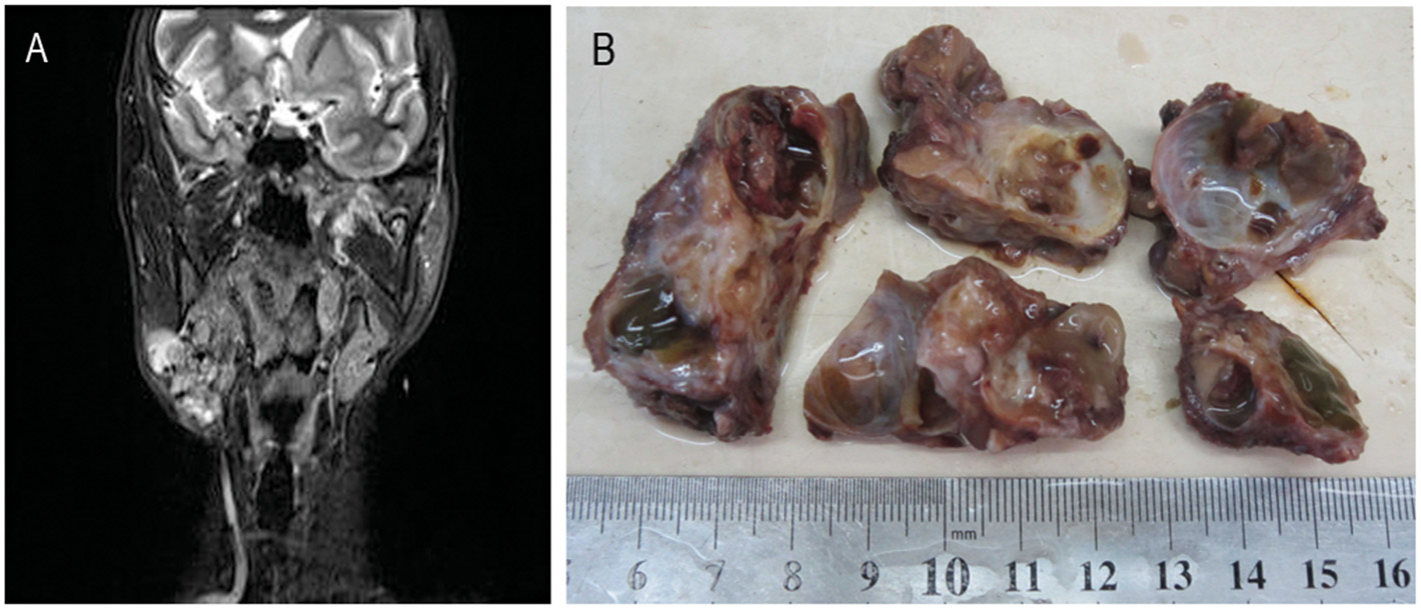
**Radiographic examination and gross appearance of the submandibular gland cystadenocarcinoma. (A)** MRI scan revealed an irregular mass in the right submandibular gland with multiple cystic regions. **(B)** The cut surface of mass exhibited a nodular structure with multiple cysts filled with mucus-like materials.

A 44-year-old man presenting with a painless mass in the right submandibular region that he reported to have for 30 years visited our clinic in August, 2013. The patient complained that the mass had enlarged rapidly in the gland area during the past three months. Physical examination revealed a hard and nodular mass (7 × 6 cm) with an unclear boundary. The temperature and color of the overlying skin was normal, with no ulcerations or hemorrhaging. The patient’s regional lymph nodes were not enlarged. A magnetic resonance imaging (MRI) scan revealed an irregular mass in the right submandibular gland with multiple cystic regions (Figure 1A). Results of routine laboratory blood and urine tests were within normal ranges. The computed tomography (CT) scan showed that the chest and abdomen appeared to be free of cancers and metastasis. The tumor was surgically excised and no metastasis or recurrence was observed during the five months of post-surgery follow-up examinations.

**Figure 2:**
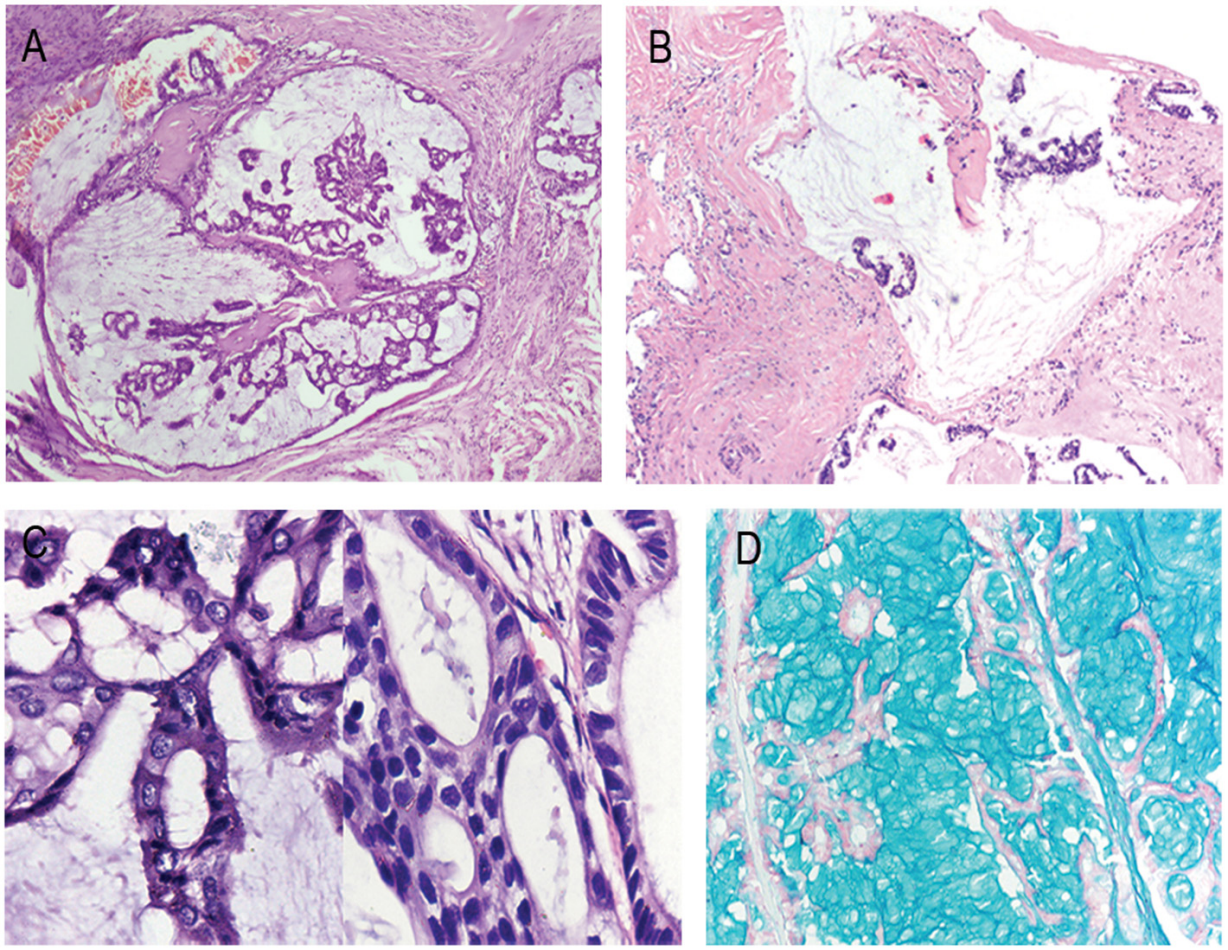
**Histopathological characteristics of the submandibular gland cystadenocarcinoma. (A)** Most of the cysts were lined by a single or multiple layers of epithelial cells that formed papillary, mesh, or cribriform architectures (×40). **(B)** Some cysts had no epithelial lining, with scattered tumor cells or cell clusters floating in the mucus lakes (×40). **(C)** The tumor cells were cuboidal or low columnar (×400). **(D)** Alcian Blue staining showed both cysts and tumor cells were filled with mucus-like materials (×100).

The tumor was encapsulated, soft, irregularly shaped, and measured 5.5 cm × 5 cm × 4 cm. The cut surface exhibited a nodular structure with multiple cysts filled with mucus-like materials (Figure 1B). Microscopically, the encapsulated mass was shown to be separated into a number of varying sized cysts by fibrous tissues. The cysts were each filled with mucus-like materials. Most of the cysts were lined by a single or multiple layers of epithelial cells that formed papillary, mesh, or cribriform architectures (Figure 2A). However, a number of cysts had no epithelial lining, with scattered tumor cells or cell clusters floating in the mucus lakes (Figure 2B). The hemorrhage and cholesterol crystals were also observed in these cysts. The atypical tumor cells were cuboidal or low columnar and characterized with eosinophilic or basophilic cytoplasm. Abundant intracellular mucus was observed in the tumor cells. The tumor cells had round or oval nuclei with uniformly distributed fine chromatin or bubble-like nuclei. Eosinophilic nucleoli without mitotic figures were observed. Tumor cells in other areas without cysts structures formed complex papillary and infiltrated into the surrounding stromal (Figure 2C).

Immunohistochemical staining showed that the tumor cells were CK, CKH, CK8/18, CK7, CK19, and S-100 positive. Approximately 2% of the cells were Ki67 positive. The cells were CK5/6, CEA, Actin, P63, Calponin, SMA, P53, CDX2, CK20, Villin, C-erbB-2, TTF1, SP-A, and TG negative. Alcian blue and Periodic Acid Schiff staining demonstrated that both cysts and tumor cells were filled with mucus-like materials (Figure 2D). Based on these observations, this tumor was diagnosed as a submandibular gland cystadenocarcinoma.

In 2005, cystadenocarcinoma of salivary glands was reclassified by the World Health Organization (WHO) as a malignant tumor that rarely occurs in submandibular glands [1]. Salivary gland cystadenocarcinomas account for approximately 2% of malignant salivary gland tumors. It is a rare tumor, compared with other tumor in the salivary gland [2,3]. Most cystadenocarcinoma have been reported in the parotid gland and minor salivary glands. Since being first described in 2006, only four cases of submandibular gland cystadenocarcinoma have been reported. Among the four cases, all patients were male, aged 12, 54, 67, and 74 years old [4-7]. Similarly, the patient in our study was a 44-year-old man. The main symptom of a submandibular gland cystadenocarcinoma is swelling in the submandibular gland region. Some patients complain of pain or tenderness, but bleeding, ulceration, and dysfunction are rare. Risk factors for cystadenocarcinoma remain unclear. However, smoking, alcohol use, environmental factors, and genetic abnormalities may contribute to the carcinogenesis. The most effective treatment is surgical excision of the tumor and most patients recover.

Based on WHO descriptions, cystadenocarcinoma is primarily characterized by a cystic structure of varying sizes, lined by epithelial cells with or without papillary projections. In the first round of biopsy and histopathology examination, while these typical features were observed, we also identified a number of cysts lacking epithelial lining that were filled with abundant mucus-like materials. Scattered single tumor cells and cell clusters floated freely in the mucous lakes. These areas were shown to have conventional morphologic features of mucinous adenocarcinoma. Due to the low incidence of submandibular gland cystadenocarcinoma and presentation of mucinous adenocarcinoma-like areas, this tumor was initially misdiagnosed as a mucinous adenocarcinoma. Following consultation with senior physicians, additional tumor tissues were examined. Generally speaking, the majority of tumor tissues presented morphologic features of cystadenocarcinoma, but limited areas had cysts lacking epithelial cell lining caused by hemorrhaging and shedding of epithelial cells. The immunohistochemical staining of the markers mentioned above revealed no significant difference between these two types of tumors because both have glandular epithelium origins. Therefore, differential diagnosis primarily depends on examination of more tissues.

## Consent

Written informed consent was obtained from the patient for publication of this case report.

## Competing interests

The authors declared that they have no competing interests.

## Authors’ contributions

JW and CZ conducted histological examinations. CZ drafted the manuscript. CC performed H&E staining, Immunohistochemical staining, and Alcian Blue and Periodic Acid Schiff staining. All authors read and approved the final manuscript.
